# Poverty, agency, and suicide: Men and women

**DOI:** 10.1192/j.eurpsy.2022.340

**Published:** 2022-09-01

**Authors:** R. Steyn

**Affiliations:** University of South Africa, Graduate School Of Business Leadership, Midrand, South Africa

**Keywords:** suicide ideation, agency, poverty, sex

## Abstract

**Introduction:**

Assumptions linking poverty with sex, associating poverty with agency, as well as connecting agency with suicide, are widespread. Women are often seen as being affected more by poverty than men. Men are frequently considered to possess more agency than women, and men are also more prone to suicide than women.

**Objectives:**

The research aims to assess if poverty, agency and suicide differences occur across sexual lines. The study will attempt to establish if a poverty-agency-suicide relationship is supported by data, and how the poverty-agency and the agency-suicide relationships are in turn influenced by sex.

**Methods:**

A cross-sectional survey design was used and interviews were conducted with 3 531 respondents. Chi-squared tests were used to calculate whether differences on poverty, agency and suicide ideation exist across sexual lines. Correlation analysis was implemented to test for the poverty-agency-suicide relationship, and regression analyses were used to test the moderating effects of sex on the poverty-agency and the agency-suicide relationships.

**Results:**

Men and women did not differ significantly on levels of poverty, agency, nor suicide ideation. Poverty did relate to agency (a negligible effect), but agency did not have an effect on suicide ideation. Sex did not moderate the poverty-agency nor the agency-suicide relationship.

**Conclusions:**

The data do not support established stereotypes nor empirical findings regarding sex differences across the poverty, agency and suicide ideation spectrums. The data also do not support the poverty-agency-suicide relationship and sex does not influence this relationship. Healthcare professionals should be aware that (well-founded) stereotypes do not necessarily materialize in all populations.

**Disclosure:**

No significant relationships.

